# Food insecurity vulnerability among domestic migrants across Cambodian provinces: a multilevel analysis

**DOI:** 10.1136/bmjph-2025-003281

**Published:** 2026-03-04

**Authors:** Maelodee Chong Armstrong, Ailiana Santosa, Paul Kowal, Heng Sopheab, Nawi Ng

**Affiliations:** 1 School of Public Health and Community Medicine, University of Gothenburg Sahlgrenska Academy, Gothenburg, Sweden; 2 National Centre for Epidemiology and Population Health, Australian National University, Canberra, Australian Capital Territory, Australia; 3 School of Public Health, National Institute of Public Health, Phnom Penh, Cambodia

**Keywords:** Community Health, Public Health, Sociodemographic Factors, Transients and Migrants, Food Insecurity, Multilevel Analysis

## Abstract

**Introduction:**

Food insecurity affects over half of Cambodia’s population, yet despite national efforts to improve equitable access to food, little is known about how migration patterns and provincial contexts shape this vulnerability. This study examines provincial variation in food insecurity in Cambodia, its association with domestic migration patterns, modification by household-head gender and the role of provincial-level contextual factors.

**Methods:**

This cross-sectional study used data from 5166 adults aged 18 and older from the 2023 World Health Survey Plus Cambodia, linked with provincial-level data across 25 provinces. A two-level multilevel model examined the association between nine domestic migration patterns and food insecurity—defined as eating less or going hungry in the last 12 months due to lack of food or inability to afford food. The model included individual-level covariates (age, gender, wealth, education, urbanisation, household size, ethnicity and marital status), province-level factors (flooding, wealth, socio-geographic zones and special economic zones) and an interaction term between household-head gender and migration patterns.

**Results:**

Two groups of domestic migrants had higher odds of experiencing food insecurity than those who never moved: rural-to-rural migrants who moved either intra-provincially (OR 1.65, 95% CI 1.15 to 2.37) or inter-provincially (OR 1.59, 95% CI 1.15 to 2.19). Household-head gender modified the association between migration and food insecurity. Food insecurity prevalence varied across provinces, and only 1.1% of this variation was attributable to province-level characteristics, with half (0.5%) explained by flooding, provincial wealth, special economic zones and socio-geographic zones. Larger group-level variation (12.0% and 9.1%) was observed at the sub-provincial district level.

**Conclusions:**

Domestic migrants moving between rural areas in Cambodia face higher risks of food insecurity. Policy-makers are encouraged to strengthen labour laws and social protection programmes, implement targeted interventions that account for migration and gendered effects and enhance data systems, especially at sub-provincial levels, to enable further research.

**WHAT IS ALREADY KNOWN ON THIS TOPIC**

- Cambodia aims to eliminate food insecurity by addressing disparities in food access and revising intervention strategies to account for layered vulnerabilities, yet little is known about how food insecurity varies across provinces, between domestic migrant groups, and how these relationships are modified by the gender of the household head.

**WHAT THIS STUDY ADDS**

- Domestic migrants who move between rural areas are more vulnerable to food insecurity, and female-headed households who move intra-provincially from rural to urban areas face higher odds than expected. Food insecurity prevalence varies across provinces, and 1.1% of this variation is attributable to province-level factors, half of which (0.5%) is explained by flooding, provincial wealth, special economic zones and socio-geographic zones. Sensitivity analyses indicate that 9.1% to 12.0% of the variation attributable to the group-level exists at sub-provincial levels such as districts.

**HOW THIS STUDY MIGHT AFFECT RESEARCH, PRACTICE OR POLICY**

- Policy-makers are encouraged to strengthen labour laws and social protection programmes, implement targeted, context-specific interventions that account for migration and gendered effects and ensure the tracking and availability of data on these factors, especially at sub-provincial levels, to support further research on how to decrease inequity and eliminate food insecurity.

## Introduction

 Food insecurity remains one of the most pressing global challenges, affecting billions of people and undermining progress toward sustainable development. An estimated 28.9% of the global population (2.3 billion people) experienced food insecurity in 2023.^1^ Food insecurity is a condition in which people do not have access to food, or food of an adequate quality, to meet their basic needs. It ranges from moderate food insecurity, where people experience uncertainty about their ability to obtain food and reduce the quality or quantity of their food consumption, to severe food insecurity, where individuals run out of food and go without eating for extended periods.^2^ Although prevalence has declined in North America and Europe, it has risen in most other regions over the past decade, with Asia experiencing the sharpest increase.

Food insecurity disproportionately affects low- and middle-income countries (LMICs). In Southeast Asia, Cambodia has the highest prevalence of severe food insecurity (13.9%) and the second highest (50.5%) when moderate and severe food insecurity are combined, following only Timor-Leste (53.7%).^1^ Despite the Cambodian government’s commitment to eliminating food insecurity and hunger,^3^,^5^ significant challenges persist. Although the country produces a surplus of rice for export,^6^ food access remains unstable due to recurring natural shocks—particularly floods—and socio-economic vulnerabilities, such as seasonal or low income and volatile food prices.^7^ These structural pressures intersect with broader shifts in livelihoods and mobility, as rural households adapt to changing economic conditions and environmental stressors.

Cambodia’s socio-economic context has steadily evolved, with a declining rural population and reduced reliance on agriculture for employment.^8^ Historically, agricultural households spent little on food, relying primarily on their own produce and livestock. However, the government’s active promotion of rice exports since 2015 has intensified rice production. Many households now grow two or three rice crops per year, reducing their ability to diversify food sources or raise livestock, making them increasingly reliant on markets.^6^

About 20% of Cambodia’s population are domestic migrants—individuals who move to another area within a country.^9^ These migration patterns have shifted significantly over the past three decades, from predominantly rural-to-rural to urban-to-urban movements^10^ and from intra-provincial to inter-provincial flows.^9^ These shifts are driven by a mix of push factors, such as poverty and climate shocks, and pull factors like employment or marriage opportunities.^6 9 11^ These changes reflect how households adapt to economic and environmental stress. While migration can be a coping strategy, its impact on food security is complex.

In other LMICs, households with migrant members have reported reduced food insecurity, increased savings and improved resilience to shocks.^12^,^14^ However, migration may also reflect pre-existing resource advantages^15^ or a ‘reverse migration’ from labour market disruptions.^16^ In Cambodia, those left behind may face increased food insecurity due to insufficient remittances,^17^ rising debt or a shift in food consumption practices such as reliance on instant noodles.^6^ Yet little is known about food insecurity among the migrants themselves. Women are disproportionately affected by climate vulnerability due to persistent inequalities in social and economic factors.^18^ Women-headed households are additionally more vulnerable to land grabbing^18^ and food insecurity^19 20^ than men-headed households.

Contextual factors may help explain why food insecurity varies across provinces and among different population groups, making them essential to understanding the lived realities of domestic migrants in Cambodia. Floods occur regularly and affect livelihoods.^5 21 22^ Provincial wealth disparities are also pronounced: no one in Phnom Penh falls into the national lowest wealth quintile, while 75% of Ratanak Kiri’s population belongs to it.^23^ Furthermore, structural processes such as national development policies, institutional norms and social hierarchies—especially those related to gender, age and rural–urban divides—also shape household livelihoods and access to food.^7 24^ One such factor is the Cambodian government’s investment in Special Economic Zones (SEZs), which are designated areas with favourable administrative regulations to attract business and investment.^25^ Greater investment opportunities can expand job availability and diversity, increasing household incomes and improving access to food.^7^

This study aims to examine (1) whether there are significant differences in food insecurity across provinces in Cambodia, (2) whether domestic migration patterns are associated with migrants’ food insecurity and whether this association is modified by household-head gender and (3) to what extent variation in food insecurity can be attributed to the provincial level and explained by contextual factors.

## Materials and methods

**Figure 1:**
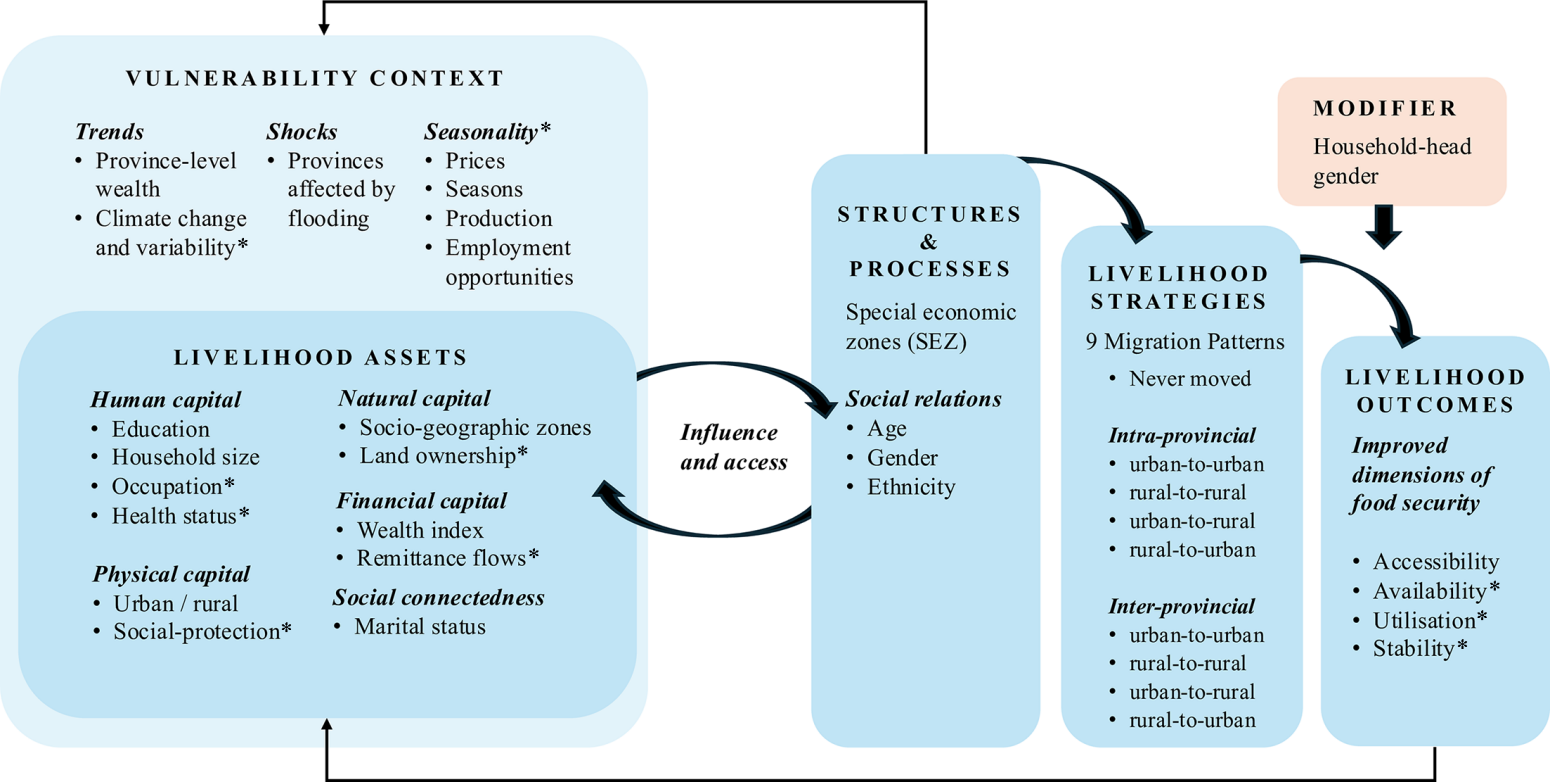
Conceptual framework. How domestic migration shapes vulnerability to food insecurity, adapted from the Sustainable Livelihoods Framework.^26^ *Variables acknowledged as part of the conceptual framework but not investigated in this study due to limitations in data availability.

To better understand how domestic migration shapes vulnerability to food insecurity, this study draws on the Sustainable Livelihoods Framework (figure 1). The framework illustrates how individuals leverage assets and strategies to pursue livelihood outcomes while operating within a context of vulnerability, structures and processes.^26^ For this study, the framework was adapted to reflect Cambodia-specific elements and replaced social capital with social connectedness due to the lack of available indicators.

### Study design and study population

This study analysed data from the World Health Survey Plus (WHS+),^27^ implemented in 2023 in Cambodia. The nationally representative dataset consists of 5275 observations collected using a three-stage cluster sampling design across all 25 provinces. In each eligible household, a household head completed a questionnaire listing all household members using a computer-assisted personal interview (CAPI). One individual aged 18 years or older was then randomly selected by the CAPI to complete the individual questionnaire and was not replaced if unavailable for any reason. The non-participation rate was approximately 13%, primarily due to refusals, ineligibility (eg, mental incapacity or illness) or respondent unavailability. All provinces included both urban and rural areas, except Phnom Penh, which consisted solely of urban areas.

### Measuring food insecurity

This study measured accessibility as a dimension of food insecurity using two questions: (1) ‘In the last twelve months, how often did you ever eat less than you felt you should because there wasn’t enough food?’ and (2) ‘In the last twelve months, were you ever hungry, but didn’t eat because you couldn’t afford enough food?’. Response options for both questions were coded on a 5-point scale: 1=every month; 2=almost every month; 3=some months, but not every month; 4=only in 1 or 2 months; 5=never. Following previous studies,^28 29^ individuals who selected options 1, 2 or 3 for both questions, or option 1 for either question, were classified as having experienced severe food insecurity. Individuals who selected 5 for both questions were classified as food secure. All remaining respondents were categorised as experiencing some food insecurity. These categories were then collapsed into a binary variable distinguishing between food secure and any food insecurity (some or severe).

### Measuring migration patterns

Individuals who had never moved were identified using the question ‘Have you always lived in this village/town/city?’. To address the potential bidirectional relationship between migration and food insecurity, the study included only individuals who had migrated before the 12-month period covered by the food insecurity questions. The question, ‘How long have you been living (continuously) in this area?’, was used to exclude individuals who had moved within the past year (n=19).

The question, ‘Where were you living before?’, was used to identify migration patterns, including the origin and destination of individuals. The question did not ask how long they had lived in their previous location. Respondents who reported having previously lived ‘outside Cambodia’ were classified as international migrants and excluded from the analysis (n=23).

Responses of ‘In the same community/locality/neighbourhood’, ‘In another city in this province’ and ‘In another rural area in this province’ were coded as intra-provincial migration, while responses ‘In another city outside this province but in Cambodia’ and ‘In another rural area outside this province but in Cambodia’ were coded as inter-provincial migration. To determine urban-rural migration patterns, these responses were cross-referenced with the reported urban or rural classification of the respondent’s current residence. This categorisation resulted in nine migration categories (figure 1).

Duration of residence after migration was determined using the question ‘How long have you been living (continuously) in this area?’. Responses were grouped into seven categories, ranging from ‘never moved’ to ‘31 years or more’ in 5-year increments.

### Measuring individual-level modifier

Household head refers to the primary decision-maker in each household. Household-head gender, based on identified gender, was coded as ‘female-headed’ or ‘male-headed’, following established research terminology. Respondents lacking information on this variable were excluded from the analysis (n=67). After excluding recent movers (n=19), international migrants (n=23) and respondents with missing values for household-head gender (n=67), the final analytic sample consisted of 5166 respondents.

### Measuring individual-level confounders

All individual-level control variables were categorical (online supplemental table S1). Age was categorised into six groups (18–29, 30–39, 40–49, 50–59, 60–69 and 70+). The highest education level achieved was categorised into five groups (never schooled, incomplete primary, completed primary, completed secondary and at least high school). Household size was a count from one to seven or more members. Marital status was included as an indicator of social connectedness, based on the assumption that individuals who are married or cohabiting are more likely to have a broader social network than those living alone. Given that food insecurity was assessed over the past 12 months, marital status was coded to reflect the individual’s status prior to this period. Specifically, individuals who had been separated, divorced or widowed for less than a year were classified as married or cohabiting, while those who had been married or cohabiting for less than a year were categorised as never married.

Physical capital refers to basic infrastructure, for example, access to transport, water and energy.^26^ In this study, it was measured by whether respondents lived in urban or rural areas, as urban populations generally have better access to water and sanitation^30^ and receive prioritisation in infrastructure development.^31 32^ The geographic variable of urban or rural location of dwelling was available as part of the sampling frame.

Gender was measured using a self-reported variable where respondents could identify as ‘man’, ‘woman’ or ‘non-binary’. As no respondents selected ‘non-binary’, this variable was treated as binary in the analysis. Ethnicity was also self-reported, based on the question ‘What is your background or ethnic group?’. For analytical purposes, responses were dichotomised (Khmer or Non-Khmer) due to the small sample sizes of minority ethnic groups.

Housing and asset variables were used to create a household wealth index, which was constructed using the Demographic and Health Survey Wealth Index guidelines.^33^ Only variables with sufficient variation in ownership (ranging between 12.5% and 87.5%) were considered for Principal Component Analysis (PCA) and assessed using the Keiser-Meyer-Olkin test. A tetrachoric correlation matrix was generated using the Stata command ‘*tetrachoric*’, and PCA was then applied using ‘*pcamat*’, followed by the ‘*rotate*’ command to extract components. The first principal component, which explained 21.1% of total variance, was used to construct the wealth index. The resulting wealth scores were ranked in ascending order and divided into quintiles. To account for differences in asset ownership patterns, PCA was conducted separately for urban and rural households using different indicators (online supplemental tables S2 and S3). The resulting urban and rural wealth indices were then combined into a single composite wealth index variable for use in the analysis.

### Measuring contextual-level confounders

Cambodian provinces were grouped into four socio-geographic zones—Coastal, Plain, Plateau and Tonle Sap Lake^34^—and were used to account for environmental shocks. Provinces affected by floods in 2022, as reported by the Humanitarian Response Forum,^35^ were coded as 1 (flooded), while the others were coded as 0 (not flooded).

Provincial wealth level was measured using the population proportion in each province that fell within the national lowest wealth quintile, as reported by the National Institute of Statistics, Ministry of Health and ICF’s report.^23^ Provinces were then categorised as ‘poorer’ or ‘richer’ based on whether the percentage was above or at/below the national median.

Economic investment was captured through the number of SEZs in each province. According to Open Development Cambodia,^36^ a total of 50 SEZs were established between 2006 and the start of the WHS+implementation (13 March 2023), based on data compiled from royal gazettes and reports from various governmental ministries. The number of SEZs per province ranged from 0 to a maximum of 12 in Preah Sihanouk (online supplemental data D1).

### Data preparation

The wealth index contained 192 missing observations, which were addressed through multiple imputation. A total of 21 imputed data frames were generated using the ‘mice’ function^37^ in R. These imputed datasets were then merged into a single data frame using the ‘merge imputations’ function from the ‘sjmisc’ package.^38^ All subsequent analyses were conducted in Stata V.18.^39^

Multicollinearity among variables was assessed using the ‘variance inflation factor’ (VIF). The mean VIF was three, with the highest value of seven. As a general rule, further investigation of multicollinearity is not required when VIF values are below ten.^40^

### Analysis

The analysis began with descriptive statistics, including duration of residence post-migration, a variable that was not incorporated into the subsequent analyses. Multilevel logistic models were then conducted, with individuals at level one nested within provinces at level two. Multilevel modelling effectively addresses clustering, where individuals from the same area tend to share similar characteristics and outcomes, while also identifying the significance of specific contexts on individual outcomes by partitioning variance at each level.^41^

First, a null two-level model (Model 0) was built with only an intercept ($\beta_{0})$) and province-level residual ($u_{0j})$) to assess whether there were significant differences in food insecurity between provinces. Model 1 was a random intercept model with the individual explanatory variable of the nine migration categories (*mig*). In Model 2, all individual-level categorical variables were added to the previous model. These included household-head gender (*headg*), an interaction term between household-head gender and migration categories (*headg#mig*), and confounders: education level (*edu*), wealth quintile (*iwealth*), urbanisation (*urban*), household size (*hhsize*), age groups (*ageg*), gender (*gender*), marital status (*marital*) and ethnicity (*ethnic*). Model 3 added contextual factors, which contained categorical variables: flooding (*flood*), provincial wealth (*pwealth*), socio-geographic zones (*sgeoz*) and one continuous variable for the number of special economic zones (*sez*).

Model 3 is written as

,$$\begin{array}{l} \log\left(\frac{\pi_{ij}}{1-\;\pi_{ij}}\right)=\;\beta_{0}+\beta_{1}mig_{ij}+\beta_{2}headg_{ij}+\beta_{3}headg_{ij}\#mig_{ij}\\ \;+\beta_{4}edu_{ij}+\beta_{5}iwealth_{ij}+\beta_{6}urban_{ij}\\ \;+\beta_{7}hhsize_{ij}+\beta_{8}ageg_{ij}+\beta_{9}gender_{ij}\\ \;+\beta_{10}marital_{ij}+\;\beta_{11}ethnic_{ij}+\;\beta_{12}flood_{j}\\ \;+\beta_{13}pwealth_{j}+\beta_{14}sgeoz_{j}+\beta_{15}sez_{j}+u_{0j} \end{array}$$

where

- *i* denotes the individual (level one)
- *j* denotes the province (level two)
- log[*π*_*ij*/(1*−π*_*ij*)] is the log-odds of experiencing food insecurity versus not experiencing for individual *i* in province *j*.

Following Model 3, pairwise tests compared the adjusted predicted probabilities of food insecurity across all pairs of migration patterns. Key measures derived from the models include the intraclass correlation coefficient (ICC), the median odds ratio (MOR) and the 80% interval odds ratio (IOR-80) following the definitions and formulas from Merlo,^42^ with the ICC estimated using the latent variable method. The IOR-80 was only reported in Model 3. Due to the lack of province-level weights, the multilevel model could not be weighted.

Two sensitivity analyses examined the effects of alternative food insecurity thresholds. In the first, Models 0 to 3 were re-estimated using an alternative binary classification of no/some versus severe food insecurity. In the second, three categories of food insecurity (no/some/severe) were analysed using a multilevel multinomial regression.

Provinces were chosen as the second-level unit based on the recommendation that each group have multiple outcome observations^43^ and to investigate province-level factors. Two more sensitivity analyses assessed differences in ICC and MOR across models 0, 1 and 2 using districts as the second level: one including all districts and the other including only districts with more than one outcome observation.

### Patient and public involvement

The study respondents were not involved in developing the research questions, outcome measures, study design or recruitment. We have no plans to disseminate the research findings to the respondents.

## Results

Table 1 displays the descriptive statistics for the respondents, stratified by gender. Among the 5166 respondents, 1584 (30.7%) were men and 3582 (69.3%) were women. More women (32.7%) belonged to female-headed households than men (8.8%). Most respondents were either currently married or cohabiting (78.0%) and Khmer (92.3%). Most respondents were aged between 30 and 59. Half of the respondents had either not completed primary education (34.8%) or had no formal schooling (21.7%), and a higher percentage of men were in the wealthier quintiles and had higher levels of education compared with women.

**Table 1:** Descriptive characteristics of the study respondents by total and gender (N, %)

	Men	Women	Total
	1584 (30.7)	3582 (69.3)	5166 (100)
Household-head gender			
Male-headed	1444 (91.2)	2409 (67.3)	3853 (74.6)
Female-headed	140 (8.8)	1173 (32.7)	1313 (25.4)
Age groups			
18–29	183 (11.6)	464 (13.0)	647 (12.5)
30–39	319 (20.1)	773 (21.6)	1092 (21.1)
40–49	355 (22.4)	713 (19.9)	1068 (20.7)
50–59	329 (20.8)	743 (20.7)	1072 (20.8)
60–69	266 (16.8)	629 (17.6)	895 (17.3)
70+	132 (8.3)	260 (7.3)	392 (7.6)
Highest education level			
At least high school	210 (13.3)	230 (6.4)	440 (8.5)
Completed secondary	241 (15.2)	382 (10.7)	623 (12.1)
Completed primary	421 (26.6)	763 (21.3)	1184 (22.9)
Incomplete primary	486 (30.7)	1314 (36.7)	1800 (34.8)
Never schooled	226 (14.3)	893 (24.9)	1119 (21.7)
Wealth index			
Q1 (poorest)	299 (18.9)	757 (21.1)	1056 (20.4)
Q2	279 (17.6)	732 (20.4)	1011 (19.6)
Q3	322 (20.3)	800 (22.3)	1122 (21.7)
Q4	317 (20.0)	680 (19.0)	997 (19.3)
Q5 (richest)	367 (23.2)	613 (17.1)	980 (19.0)
Area residence			
Urban	651 (41.1)	1363 (38.1)	2014 (39.0)
Rural	933 (58.9)	2219 (61.9)	3152 (61.0)
Household size			
1	34 (2.1)	78 (2.2)	112 (2.2)
2	169 (10.7)	361 (10.1)	530 (10.3)
3	253 (16.0)	541 (15.1)	794 (15.4)
4	411 (25.9)	914 (25.5)	1325 (25.6)
5	327 (20.6)	742 (20.7)	1069 (20.7)
6	187 (11.8)	479 (13.4)	666 (12.9)
7+	203 (12.8)	467 (13.0)	670 (13.0)
Marital status before past 12 months			
Currently married or cohabiting	1360 (85.9)	2669 (74.5)	4029 (78.0)
Never married	119 (7.5)	193 (5.4)	312 (6.0)
Separated/divorced/widowed	105 (6.6)	720 (20.1)	825 (16.0)
Ethnicity			
Khmer	1452 (91.7)	3314 (92.5)	4766 (92.3)
Non-Khmer	132 (8.3)	268 (7.5)	400 (7.7)

35% of respondents had migrated domestically, with the most common patterns being inter-provincial rural-to-rural migration (8.4%), inter-provincial rural-to-urban migration (7.4%) and intra-provincial rural-to-rural migration (6.4%) (table 2). Women and non-Khmer individuals were more likely to have never moved compared with men and Khmer individuals. Inter-provincial rural-to-urban migrants were generally older, while intra-provincial rural-to-urban migrants and non-migrants were generally poorer. Only 4.3% of respondents had moved between 1 and 5 years ago. Food insecurity was more prevalent among women and lower wealth quintiles and less prevalent among those aged 70 and older (online supplemental table S4).

**Table 2:** Description of migration patterns and duration of residence post-migration by gender, ethnicity, age and wealth quintile

	Total, N (%)	Gender, N (%)		Ethnicity N, (%)		Age Mean (SD)	Poorest quintile, %	Richest quintile, %
		Men	Women	Khmer	Non-Khmer			
Total	5166	1584	3582	4766	400	47.3 (14.9)	20.4	19.0
Never moved	3332 (64.5)	888 (56.1)	2444 (68.2)	3026 (63.5)	306 (76.5)	46.6 (15.2)	22.4	15.9
Migration patterns of respondents who had moved…								
Intra-provincially								
Urban-Urban	94 (1.8)	34 (2.1)	60 (1.7)	90 (1.9)	4 (1.0)	46.6 (15.2)	11.7	34.0
Rural-Rural	330 (6.4)	139 (8.8)	191 (5.3)	306 (6.4)	24 (6.0)	46.9 (13.3)	17.6	18.2
Urban-Rural	81 (1.6)	37 (2.3)	44 (1.2)	78 (1.6)	3 (0.8)	47.6 (14.7)	14.8	30.9
Rural-Urban	174 (3.4)	72 (4.5)	102 (2.8)	164 (3.4)	10 (2.5)	46.0 (15.7)	23.6	16.1
Inter-provincially								
Urban-Urban	197 (3.8)	76 (4.8)	121 (3.4)	192 (4.0)	5 (1.2)	49.3 (14.5)	11.7	35.0
Rural-Rural	436 (8.4)	143 (9.0)	293 (8.2)	413 (8.7)	23 (5.8)	48.9 (13.6)	17.4	24.8
Urban-Rural	142 (2.8)	52 (3.3)	90 (2.5)	135 (2.8)	7 (1.8)	46.2 (14.5)	14.8	35.9
Rural-Urban	380 (7.4)	143 (9.0)	237 (6.6)	362 (7.6)	18 (4.5)	50.3 (14.1)	17.6	20.8
Duration of residence for respondents who had moved more than a year ago								
1–5 years	223 (4.3)	82 (5.2)	141 (3.9)	214 (4.5)	9 (2.2)	37.2 (13.8)	17.0	25.1
6–10 years	243 (4.7)	104 (6.6)	139 (3.9)	229 (4.8)	14 (3.5)	40.1 (12.7)	17.7	28.4
11–15 years	228 (4.4)	95 (6.0)	133 (3.7)	224 (4.7)	4 (1.0)	42.1 (10.7)	13.6	24.1
16–20 years	247 (4.8)	86 (5.4)	161 (4.5)	233 (4.9)	14 (3.5)	44.7 (12.3)	17.4	26.7
21–25 years	227 (4.4)	81 (5.1)	146 (4.1)	213 (4.5)	14 (3.5)	50.9 (12.0)	20.3	20.3
26–30 years	197 (3.8)	78 (4.9)	119 (3.3)	188 (3.9)	9 (2.2)	53.2 (10.6)	13.2	23.9
31 or more years	469 (9.1)	170 (10.7)	299 (8.3)	439 (9.2)	30 (7.5)	60.7 (9.3)	17.5	24.1

### Fixed-effect analyses

**Figure 2:**
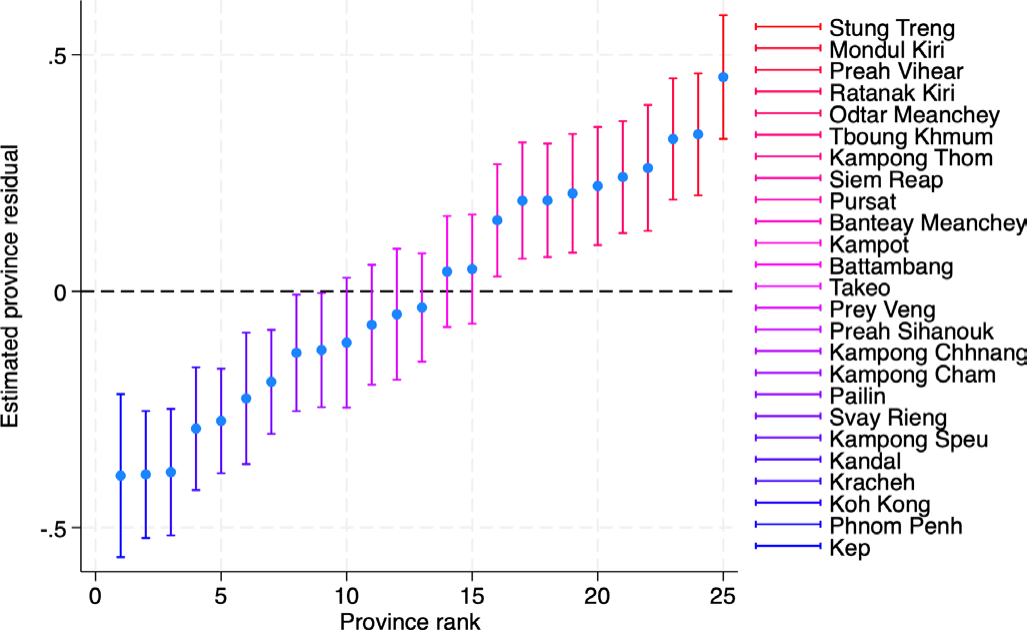
Caterpillar plot of the null model showing significant differences in food insecurity prevalence between provinces. The dotted line at 0 represents the overall intercept.

The null model (figure 2) shows significant differences in food insecurity prevalence between provinces. In most cases, the 95% CIs for provincial intercepts do not overlap with the overall mean intercept—indicating that the between-province variance is not zero (with a likelihood ratio test statistic of 49.1 and p value<0.00005) and thus warrants a multilevel model.

The individual-level fixed effects in Model 3 (table 3) show that rural-to-rural migration is associated with higher odds of experiencing food insecurity—regardless of whether the move was within the same province (OR 1.65, 95% CI 1.15 to 2.37) or to another province (OR 1.59, 95% CI 1.15 to 2.19). Female-headed households were more likely (OR 1.23, 95% CI 1.08 to 1.40) to experience food insecurity than male-headed households. The ORs for the contextual factors were not statistically significant. Pairwise differences in adjusted predicted probabilities across migration patterns ranged from −7.4% to −21.2% for comparisons indicating lower predicted probability of food insecurity and from 9.6% to 19.1% for comparisons indicating higher probability (online supplemental table S5).

**Table 3:** Associations between domestic migration patterns and household-head gender with food insecurity

	Model 0	Model 1	Model 2	Model 3
Respondents	5166	5166	5166	5166
Measures	OR (95% CI)	OR (95% CI)	OR (95% CI)	OR (95% CI) (IOR-80)
**Fixed effects at the individual level**				
Intra-provincial migration *(ref: never moved)*				
Urban-Urban		0.86 (0.53 to 1.42)	1.63 (0.91 to 2.91)	1.62 (0.91 to 2.88)
Rural-Rural		1.50* (1.07 to 2.09)	1.65** (1.14 to 2.37)	1.65** (1.15 to 2.37)
Urban-Rural		0.91 (0.53 to 1.54)	1.2 (0.74 to 1.95)	1.19 (0.73 to 1.94)
Rural-Urban		1.40* (1.04 to 1.88)	1.43* (1.00 to 2.05)	1.42 (0.99 to 2.04)
Inter-provincial migration *(ref: never moved)*				
Urban-Urban		0.37^***^ (0.23 to 0.62)	0.68 (0.41 to 1.12)	0.68 (0.41 to 1.13)
Rural-Rural		1.45* (1.03 to 2.04)	1.55* (1.11 to 2.16)	1.59** (1.15 to 2.19)
Urban-Rural		0.88 (0.61 to 1.28)	1.21 (0.81 to 1.83)	1.23 (0.82 to 1.84)
Rural-Urban		0.82 (0.63 to 1.07)	1.03 (0.78 to 1.35)	1.04 (0.79 to 1.36)
Female-headed household (*ref: male-headed*)			1.23** (1.09 to 1.40)	1.23** (1.08 to 1.40)
**Fixed effects at the province level**				
Flooding *(ref: no flooding)*				1.07 (0.85 to 1.35) (0.84–1.37)
Poorer province *(ref: richer)*				1.21 (0.91 to 1.61) (0.95–1.55)
Socio-geographic zones *(ref: coastal)*				
Plain				1.22 (0.85 to 1.77) (0.96–1.56)
Plateau				1.13 (0.71 to 1.81) (0.89–1.45)
Tonle Sap				1.03 (0.67 to 1.58) (0.80–1.31)
SEZ				1.00 (0.96 to 1.03) (0.78–1.27)
Constant	0.82 (0.72 to 0.93)	0.81 (0.69 to 0.95)	0.17 (0.12 to 0.24)	0.13 (0.09 to 0.21)
**Random effects**				
ICC	2.3% (1.3% to 3.7%)	2.3% (1.4% to 3.9%)	1.1% (0.4% to 2.8%)	0.6% (0.2% to 1.9%)
MOR	1.30 (1.21 to 1.39)	1.30 (1.21 to 1.40)	1.20 (1.09 to 1.30)	1.14 (1.05 to 1.23)

***p<0.001, **p<0.01, *p<0.05.

Model 0 was the null model with no explanatory variable. Subsequent models build upon it and each other by adding more factors. Model 1 included migration patterns. Model 2 added an interaction term between migration patterns and household-head gender and adjusted for confounders: education, individual wealth, urbanisation, household size, age, gender, marital status and ethnicity. Model 3 added province-level factors such as flooding, provincial wealth, socio-geographic zones and SEZs.

ICC, interclass correlation; IOR-80, interval odds ratio at 80%; MOR, median odds ratio; SEZ, special economic zone.

The alternative binary sensitivity analysis confirmed the main analyses’ results for rural-to-rural migrants (online supplemental table S7). An additional finding was that intra-provincial urban-to-urban migrants had higher odds of severe food insecurity (OR 2.00, 95% CI 1.20 to 3.35). Similar findings were observed in the multinomial analysis (online supplemental table S7).

Household-head gender modified the relationship between migration patterns and food insecurity, with a positive interaction observed for female-headed households migrating intra-provincially from rural to urban areas, resulting in higher odds than expected based on household-head gender and migration pattern (OR 3.69, 95% CI 1.68 to 8.10) (online supplemental table S8). Predicted probabilities are reported in online supplemental table S9.

### Random effect analyses

The ICC, which reflects the percentage variation in food insecurity attributable to the province level, was 2.3% in Models 0 and 1. The reduction to 1.1% in Model 2 indicates that approximately half of the provincial-level variation is explained by the model’s individual-level factors. In Model 3, the ICC further drops to 0.6%, suggesting that province-level contextual variables account for an additional portion of the variation. However, 0.5% of the variation remains unexplained at the province level. The MOR in Model 3 is 1.14.

Sensitivity analyses of Model 2 using districts as a second-level unit revealed a higher ICC (12.0%) and MOR (1.89) and an ICC of (9.1%) and MOR (1.73) when restricted to districts with more than one outcome observation (online supplemental table 10). Using the alternative binary classification of food insecurity resulted in higher ICCs for Models 0–3, at 5.8%, 6.0%, 4.3% and 1.8%, respectively (online supplemental table S6).

## Discussion

This study examined regional variation in food insecurity in Cambodia, its association with domestic migration patterns and the role of provincial-level contextual factors. The main findings revealed significant variation in food insecurity prevalence across provinces. Domestic migrants who move between rural areas have higher odds of experiencing food insecurity compared with non-migrants, regardless of whether moving within the same province or to a new province. Household-head gender modified this association. Most of the variation is attributed to individual-level characteristics, with only 1.1% of the variation being linked to province-level factors, half of which (0.5%) was explained by flooding, provincial wealth, socio-geographic zones and SEZs. By identifying which domestic migrants and which provinces are more vulnerable to food insecurity, this study contributes to Cambodia’s national aim of eliminating food insecurity by addressing disparities in food access, revising intervention strategies to account for proportional, absolute and layered vulnerabilities and strengthening social safety nets.^3 4^

Individuals who move between rural areas may experience a heightened risk of food insecurity due to the nature of their livelihoods and broader structural vulnerabilities in rural Cambodia. Most rural Cambodians engage in agriculture,^44^ suggesting that many rural-to-rural migrants seek wage employment on other farms, where their livelihoods remain susceptible to natural shocks and market volatility. Climate hazards vary regionally, with flooding along the Mekong River Basin, drought in the southeast, sea-level rise in coastal areas and heat stress in urban areas.^22^ Still, flooding remains the most prevalent and recurring climate hazard in Cambodia^5^ and is projected to intensify due to climate change, which reduces crop yields, particularly for wet-season rice, which is more sensitive to rainfall variability than irrigated dry-season rice.^22^ Future research should consider climate hazard variability and seasonality.

Additional structural vulnerabilities may include weak land property rights in rural areas^44^ and distress land sales to cover healthcare costs and debt.^45^ Previous studies in Cambodia highlight how agrarian intensification,^6 24^ indebtedness,^6 45^ land loss^6 24^ and limited access to social protection^7 46^ influence livelihood precarity and food insecurity among rural populations. Although this study did not directly measure these factors, its findings are interpreted within this broader socio-political context. Rural-origin domestic migrants who have experienced such hardships prior to migrating likely carry these vulnerabilities into their new settings. This study represents the first nationally representative analysis of this issue, and future mixed-method research is recommended to deepen understanding of these dynamics.

The strong positive interaction observed for female-headed households migrating intra-provincially from rural to urban areas highlights important intersectional effects. Livelihood strategies in Cambodia are highly gendered as women bear primary responsibility for securing household food and, when access is limited, are more likely than men to reduce their intake, borrow food or seek assistance from friends and neighbours.^24^ Urban labour markets are similarly gender-segmented, with women concentrated in the garment sector and men in construction.^11 47^ Gender stereotypes further shape job allocation and wage disparities, as women in construction are typically restricted to low-skilled tasks, while men in garment factories disproportionately occupy technical and managerial roles. This segregation extends to unions, where women are under-represented and frequently confined to administrative roles.^47^ Prevailing social norms reinforce these inequalities by expecting women to be compliant at work^47^ and contributing to time poverty due to their primary childcare responsibilities.^18^

The higher odds of severe food insecurity among individuals who moved intra-provincially between urban areas indicate greater vulnerability within this group. Although Cambodia’s *National Social Protection Policy Framework*^48^ reports that 41% of the population is covered by social health protection schemes and that its social assistance programmes provide cash transfers to vulnerable households, it also acknowledges that many informal workers remain unregistered in the social security schemes and therefore do not receive the intended benefits. Challenges persist in inadequate social protection^46^ and weak labour law implementation, particularly in SEZs, which, despite creating jobs, have been criticised for restricting the workers’ freedom of expression.^25^

Migrants often carry debt from rural households—often tied to microfinance loans—and may be pushed into risky or bonded labour arrangements that perpetuate financial insecurity.^49^ Beyond strengthening labour law implementation and social protection, policies and research could support social networks for domestic migrants. Positive social networks among coworkers can serve as crucial channels to increase awareness of rights and offer support in cases of rights infringements.^50^ However, many Cambodian domestic migrants, particularly those sending remittances, lack disposable income for social activities, which limits integration and access to networks.^51^ Expanding free community spaces and targeted social programmes for domestic migrants could facilitate networking opportunities. Further qualitative studies exploring the lived experiences of domestic migrants would offer deeper insights into how factors such as pay regularity and adequacy of social safety nets shape food access and employment security.^52^

Although flooding was expected to be a key driver of food insecurity at the provincial level, Model 3 reveals that its impact was not more significant than that of provincial wealth, socio-geographic zones and SEZs. These results support current policy priorities that aim to improve resilience to natural shocks but also to expand access to healthcare, social assistance and economic opportunities.^5 53^ This could include expanding access and coverage of the national social assistance programme and social security scheme.^46 48^ Estimating and disaggregating programme coverage by provincial and sub-provincial levels could help clarify this variation and better inform targeted interventions.

The share of variation attributable to the province-level (1.1%) was minimal compared with the individual-level, after adjusting for individual-level characteristics. Nevertheless, individuals living in higher-risk provinces had a 14% greater risk, in median, compared with those with similar characteristics in lower-risk provinces. When districts were used as the second-level unit instead of provinces, the variation attributable to the district-level was substantially higher (12.0% and 9.1%), indicating that meaningful contextual variation and greater increase in risk may exist at sub-provincial geographic levels. Therefore, if larger datasets are available, future research should consider exploring food insecurity variation at sub-provincial levels. Furthermore, contextual factors such as climate change risk, employment opportunities and access to social protection should be made available at sub-provincial levels. If longitudinal data were accessible, analyses incorporating additional contextual factors, such as seasonal variation and market volatility,^6^ return migration due to shifting international policies^17^ and technological trends^11^ could offer deeper insights into disparities in food insecurity across Cambodia.

This underscores the importance of targeted, context-specific interventions over a one-size-fits-all national approach, in line with Cambodia’s *Roadmap for Food Systems for Sustainable Development 2030*,^53^ which promotes inclusive food governance through provincial and municipal working groups. With 25 such groups meeting quarterly,^4^ this study encourages sharing sub-provincial data on actions and outcomes to enable future research assessing the effectiveness of localised efforts in promoting equity. Recent policies, including the *Third National Strategy for Food Security and Nutrition 2024–2028 (3rd NSFSN*)^4^ and *Third Nationally Determined Contribution (NDC 3.0*),^5^ aim to ensure equitable food access and climate adaptability for women, diverse gender groups, children and youth, indigenous peoples and people with disabilities. Our findings indicate that domestic migrants who move between rural areas should also be considered and consulted in future discussions of equity.

### Strengths and limitations

This study is the first to investigate the relationship between domestic migration and the migrants’ experiences of food insecurity in Cambodia. The data are recent, with the WHS+being collected in 2023 from a population-based sample. The inclusion of contextual data from multiple publicly available sources enhances transparency. Furthermore, the use of the multilevel method helps to shed light on the variation of food insecurity across provinces and the influence of contextual factors. The risk of recall bias is mitigated by not restricting the migration period to a specific timeframe. Additionally, robust standard errors were estimated using Stata’s ‘*vce(robust*)’ option to address potential heteroscedasticity.

Several limitations should be noted regarding inference, generalisability and the variables’ availability. First, the cross-sectional design prevents causal inference, limiting interpretation to associations. Being unable to use weights in the model may limit the generalisability of the findings. Although the study assessed food insecurity that occurred after migration, the potential bidirectional relationship between migration and food insecurity suggests that respondents may have also experienced food insecurity prior to moving. Including variables such as self-rated health, land ownership and health insurance would have allowed for a more comprehensive assessment, but these variables were not measured prior to food insecurity and were therefore excluded. Furthermore, data on occupation, remittance flows, reason for migration and access to social protection were unavailable, although these variables may confound the relationship with food insecurity. Additionally, this study assumes that individuals (n=28) who reported being married or cohabiting for less than a year had never been married, though some may have recently re-partnered after separation, divorce or widowhood.

Lastly, Cambodia’s food security framework^4 53^ encompasses dimensions beyond the scope of this study. Although this analysis focuses on the accessibility dimension of food insecurity, the framework also addresses food availability, through productivity, diversification and reserves; food utilisation, by reducing diet-related non-communicable diseases; and food stability, by enhancing resilience to climate change and disasters. By concentrating on accessibility, this study may overlook broader systemic factors that influence food availability, utilisation and the long-term stability of all three dimensions.^1^

## Conclusion

This study highlights domestic migrant groups in Cambodia who face higher odds of experiencing food insecurity compared with non-migrants, particularly migrants who move between rural areas. Household-head gender modified the relationship between domestic migration and food insecurity. The variation in food insecurity attributable to the province level was small, with half explained by contextual factors such as flooding, provincial wealth, SEZs and socio-geographic zones. Larger variation attributed to the group-level was observed at the sub-provincial district level, indicating a need for further investigation at sub-provincial levels. Policy-makers should strengthen labour laws and social protection programmes, implement targeted context-specific interventions that account for migration and gendered effects and ensure the tracking and availability of data on these factors, especially at sub-provincial levels, to support further research. Qualitative studies are needed to explore the lived experiences of these migrants to better understand their differing vulnerabilities and to explore between-group variation at sub-provincial levels.

## Supplementary material

10.1136/bmjph-2025-003281 online supplemental file 1

10.1136/bmjph-2025-003281 online supplemental file 2

## Data Availability

Data are available upon reasonable request.
