# Supplementary material for: Food insecurity vulnerability among domestic migrants across Cambodian provinces: a multilevel analysis
Source: BMJ Public Health. 2026 Mar 4;4(1):e003281. doi: 10.1136/bmjph-2025-003281 (PMC12970070; doi:10.1136/bmjph-2025-003281)
Supplement: online supplemental file 1 [file bmjph-4-1-s001.docx]

### Supplementary Tables (online)

**Table S1 Definitions and measurements of variables**

| **Variables** | **Original variables: description** | **Variables’ measurement** |
| --- | --- | --- |
| **Dependent variable** | | |
| Food insecurity | **Q3307**. In the last 12 months, how often did you ever eat less than you felt you should because there wasn’t enough food?  **Q3308**. In the last 12 months, were you ever hungry, but didn’t eat because you couldn’t afford enough food? | 0: Food secure (never ate less or went hungry in the last 12 months)  1: Food insecure (ate less or went hungry in the last 12 months) |
| **Independent variable (Individual level)** | | |
| Domestic migration pattern | **Q1015**. Have you always lived in this village/town/city?  [If Q**1015** = Yes]  **Q1016**. How long have you been living (continuously) in this area?  [If Q**1016** = “00” meaning less than a year, responses were removed (n=19)]  [If Q**1016** ≥ 1]  **Q1017**. Where were you living before? – responses are distinguished between urban / rural areas, provinces, and country  [If Q**1017** responses included “outside Cambodia”, responses were removed (n=23)]  **Q0104**. Current residential area: urban / rural | 0: Never moved  1: Intra-provincial urban-to-urban  2: Intra-provincial rural-to-rural  3: Intra-provincial urban-to-rural  4: Intra-provincial rural-to-urban  5: Inter-provincial urban-to-urban  6: Inter-provincial rural-to-rural  7: Inter-provincial urban-to-rural  8: Inter-provincial rural-to-urban |
| **Independent variables (Contextual level)** | | |
| Flooding | The provinces reported to be affected by floods in 2022. ^35^ | 0: No flood  1: Flooded |
| Provincial wealth | Population percentages within the lowest national wealth quintile for each province were obtained from the Cambodia Demographic and Health Survey (2021–22 CDHS). ^23^ The median of these percentages was then used to group provinces into binary categories of ‘poorer’ (if their percentage was higher than the median) or ‘richer’ (if their percentage was lower than or at the median) | 0: Richer provinces (percentage of people in the lowest national wealth quintile is lower than or at the median across provinces)  1: Poorer provinces (percentage of people in the lowest national wealth quintile is higher than the median across provinces) |
| Socio-geographic zones | Groupings are taken from Cambodia Agricultural Survey 2020. ^34^ | 1: Coastal  2: Plain  3: Plateau  4: Tonle Sap |
| Special economic zones (SEZ) | 50 special economic zones recorded in Cambodia between 2006 and the beginning of the WHS+ survey completion period, which is 13 March 2023. ^36^ | Continuous variable from 0 to 12 |
| **Modifier variable (Individual level)** | | |
| Household-head gender | The following questions belong to the household roster section, which refers to recorded individuals living at the household^ as ‘%rostertitle%’ in the questionnaire. Head of household was defined as the main decision maker in the household, which can be either male or female. If two people were equal decision-makers, the older person was selected.  **Q0404.** What is “%rostertitle%’s relationship to the household head?  **Q0405.** To which gender does %rostertitle% most identify?  ^A household was defined as those who share meals (‘eat out of the same pot’) and usually stay there for at least six months a year, including people who presently may be in an institution due to their health (for example, in hospital or old people’s home) for a short time. | 0: Male-headed household  1: Female-headed household |
| **Sociodemographic variables** (**Individual confounders**) | | |
| Age groups | **Q1006.** How old are you now? | 0: 18–29  1: 30–39  2: 40–49  3: 50–59  4: 60–69  5: 70+ |
| Gender | **Q1004**. Record sex of the respondent | 0: Men  1: Women |
| Ethnicity | **Q1013**. What is your background or ethnic group? | 0: Khmer  1: Non-Khmer |
| Education level | **Q1011**. What is the highest level of education that you have completed? | 0: At least high school  1: Completed secondary school  2: Completed primary school  3: Incomplete primary  4: No formal schooling |
| Household size | **Q0401**. Total number of people in household | 1: 1 person  2: 2 people  3: 3 people  4: 4 people  5: 5 people  6: 6 people  7: 7+ people |
| Household economic group | Wealth index was created based on housing conditions (**Q0501– Q0557**) and assets indicator (**Q0701–Q0722**).    Housing conditions and asset indicators were selected as preliminary variables based on the Demographic and Health Survey (DHS) Wealth Index guidelines, such as residential’s ownership status, materials of walls, floors, and roofs, cooking fuel type, shared toilet use, toilet location, drinking water source, handwashing facilities, and asset ownership (e.g., TV, motorcycle, car, electricity). The Kaiser–Meyer–Olkin (KMO) test was applied to assess the suitability of the selected variables for Principal Component Analysis (PCA). The wealth index scores from the first principal component were ranked in ascending order and divided into five equal groups (quintiles), from Q1 (poorest) to Q5 (wealthiest) | 1: Q1 (poorest)  2: Q2  3: Q3  4: Q4  5: Q5 (wealthiest) |
| Residence area | **Q0104**. Setting (in household questionnaire) | 1: Urban  2: Rural |
| Marital status before the  past 12 months | **Q1007**. What is your current marital status?  **Q1008**. For how many years have you been separated, divorced or widowed?  [If Q**1008** = “0” meaning less than a year, respondents were categorized under currently married or cohabiting (n=50)]  **Q1009**. For how many years have you been married or living together?  [If Q**1009** = “0” meaning less than a year, respondents were categorized under never married (n=28)] | 0: Currently married or cohabiting  1: Never married  2: Separated/divorced/widowed |
| **Sociodemographic variables** (**Individual confounder – only used for descriptive characteristics in Table 2, not Models 0-3**) | | |
| Duration of residence | **Q1016**. How long have you been living (continuously) in this area? | 0: Never moved  1: 1-5 years  2: 6-10 years  3: 11-15 years  4: 16-20 years  5: 21-25 years  6: 26-30 years  7: 31 years or more |

**Table S2 Final variables included in PCA (urban)**

| **Code** | **Variables** | **Component 1 factor loadings** | **Proportion of respondents** |
| --- | --- | --- | --- |
| q0701 | Television | 0.1681 | 58.9% |
| q0703 | Car | 0.3326 | 27.5% |
| q0705 | Bicycle | 0.0229 | 56.6% |
| q0707 | Hot water | 0.2654 | 15.6% |
| q0708 | Washing machine | 0.3439 | 27.7% |
| q0710 | Refrigerator | 0.3324 | 50.3% |
| q0714 | Computer | 0.3007 | 17.0% |
| q0715 | Radio | -0.0784 | 12.9% |
| q0716 | Livestock | -0.096 | 50.3% |
| q0717 | Internet | 0.3033 | 17.8% |
| q0718 | Air-conditioning | 0.3494 | 15.7% |
| q0721 | Land | 0.0311 | 50.9% |
| q0722 | Houses | 0.2178 | 15.6% |
| q0516_1 | Toilet in own home | 0.2673 | 51.5% |
| q0519_1 | Fixed hand wash in own dwelling | 0.2608 | 43.6% |
| q0531_1_6 | Liquefied petroleum gas (LPG) / cooking gas stove | 0.2319 | 58.6% |

**Table S3 Final variables included in PCA (rural)**

| **Code** | **Variables** | **Component 1 factor loadings** | **Proportion of respondents** |
| --- | --- | --- | --- |
| q0701 | Television | 0.1681 | 42.8% |
| q0702 | Motorcycle | 0.2649 | 87.5% |
| q0704 | Electricity | 0.3408 | 83.8% |
| q0705 | Bicycle | 0.0421 | 53.1% |
| q0710 | Refrigerator | 0.4302 | 22.4% |
| q0715 | Radio | -0.0472 | 12.7% |
| q0716 | Livestock | -0.1659 | 73.9% |
| q0721 | Land | -0.0715 | 78.0% |
| q0722 | Houses | 0.0202 | 13.8% |
| q0703 | Car | 0.345 | 11.5% |
| q0708 | Washing machine | 0.4036 | 9.7% |
| q0712 | Mobile phone | 0.2694 | 95.6% |
| q0713 | Video player | 0.1487 | 7.4% |
| q0717 | Internet | 0.3463 | 5.3% |
| q0515_1 | Private toilet | -0.1025 | 81.2% |
| q0516_1 | Toilet in own home | 0.0678 | 28.7% |
| q0519_1 | Fixed hand wash in own dwelling | -0.0256 | 25.9% |
| q0531_1_6 | Liquefied petroleum gas (LPG)/ cooking gas stove | 0.2259 | 33.8% |

**Table S4 Food insecurity prevalence by province, gender, age, individual wealth quintiles, and ethnicity**

|  | Total, N | Prevalence,  N (%) |  |  | Prevalence,  N (%) |
| --- | --- | --- | --- | --- | --- |
| *National & Provinces* |  |  |  | *Age group* |  |
| National (overall) | 5,166 | 2,334 (45.2) |  | 18-29 | 293 (45.3) |
| Stung Treng | 185 | 110 (59.5) |  | 30-39 | 489 (44.8) |
| Mondul Kiri | 189 | 105 (55.6) |  | 40-49 | 494 (46.3) |
| Preah Vihear | 192 | 106 (55.2) |  | 50-59 | 500 (46.6) |
| Ratanak Kiri | 174 | 93 (53.4) |  | 60-69 | 393 (43.9) |
| Otdar Meanchey | 233 | 122 (52.4) |  | 70+ | 165 (42.1) |
| Kampong Thom | 204 | 106 (52.0) |  |  |  |
| Tboung Khmum | 202 | 104 (51.5) |  | *Wealth quintile* |  |
| Pursat | 212 | 108 (50.9) |  | Q1 (poorest) | 627 (59.4) |
| Siem Reap | 226 | 115 (50.9) |  | Q2 | 553 (54.7) |
| Banteay Meanchey | 232 | 115 (49.6) |  | Q3 | 539 (48.0) |
| Kampot | 250 | 116 (46.4) |  | Q4 | 388 (38.9) |
| Battambang | 240 | 111 (46.3) |  | Q5 (richest) | 227 (23.2) |
| Takeo | 257 | 113 (44.0) |  |  |  |
| Prey Veng | 159 | 69 (43.4) |  | *Ethnicity* |  |
| Preah Sihanouk | 201 | 86 (42.8) |  | Khmer | 2121 (44.5) |
| Kampong Cham | 164 | 68 (41.5) |  | Non-Khmer | 213 (53.3) |
| Pailin | 228 | 94 (41.2) |  |  |  |
| Kampong Chhnang | 217 | 89 (41.0) |  | *Migration pattern* |  |
| Kampong Speu | 289 | 114 (39.4) |  | Never moved | 1,496 (44.9) |
| Svay Rieng | 162 | 61 (37.7) |  | *Intra-provincial* |  |
| Kandal | 291 | 108 (37.1) |  | Urban-Urban | 37 (39.4) |
| Kracheh | 197 | 71 (36.0) |  | Rural-Rural | 184 (55.8) |
| Koh Kong | 187 | 62 (33.2) |  | Urban-Rural | 34 (42.0) |
| Phnom Penh | 185 | 61 (33.0) |  | Rural-Urban | 91 (52.3) |
| Kep | 90 | 27 (30.0) |  | *Inter-provincial* |  |
|  |  |  |  | Urban-Urban | 47 (23.9) |
| *Gender* |  |  |  | Rural-Rural | 232 (53.2) |
| Men | 1,584 | 663 (41.9) |  | Urban-Rural | 59 (41.5) |
| Women | 3,582 | 1,671 (46.6) |  | Rural-Urban | 154 (40.5) |

**Table S5 Pairwise differences in adjusted predicted probabilities (%) of food insecurity across migration patterns from Model 3**

| **Migration patterns** |  |  |  |  |  |  |  |  |  |
| --- | --- | --- | --- | --- | --- | --- | --- | --- | --- |
| Never moved |  |  |  |  |  |  |  |  |  |
| Intra-provincial Urban-Urban | 5.1 |  |  |  |  |  |  |  |  |
| Intra-provincial Rural-Rural | **10.3**** | 5.2 |  |  |  |  |  |  |  |
| Intra-provincial Urban-Rural | 2.1 | -3.0 | -8.2 |  |  |  |  |  |  |
| Intra-provincial Rural-Urban | **11.8***** | 6.7 | 1.5 | 9.7 |  |  |  |  |  |
| Inter-provincial Urban-Urban | -9.4 | -14.5 | **-19.7***** | -11.5 | **-21.2***** |  |  |  |  |
| Inter-provincial Rural-Rural | **9.6*** | 4.6 | -0.6 | 7.6 | -2.1 | **19.1**** |  |  |  |
| Inter-provincial Urban-Rural | 2.2 | -2.9 | -8.1 | 0.1 | -9.6 | 11.6 | **-7.4**** |  |  |
| Inter-provincial Rural-Urban | 2.1 | -3.0 | -8.2 | 0.0 | **-9.7*** | **11.5**** | -7.6 | -0.1 |  |
|  | Never moved | **Other migration patterns for comparison** | | | | | | | |
|  |  | Intra-provincial | | | | Inter-provincial | | | |
|  |  | Urban-Urban | Rural-Rural | Urban-Rural | Rural-Urban | Urban-Urban | Rural-Rural | Urban-Rural | Rural-Urban |

**** p<0.001, ** p<0.01, * p<0.05; Model 3 includes individual-level factors of migration patterns and their interaction with household-head gender, individual-level confounders: education, individual wealth, urbanisation, household size, age, gender, marital status, and ethnicity, and province-level factors: flooding, provincial wealth, socio-geographic zones, and special economic zones.*

**Table S6 Sensitivity analysis of associations between domestic migration patterns and household-head gender with food insecurity – using alternate dichotomisation of food insecurity of individuals who had no or some food insecurity versus severe food insecurity**

|  | Model 0 | Model 1 | Model 2 | Model 3 |
| --- | --- | --- | --- | --- |
| Respondents | 5,166 | 5,166 | 5,166 | 5,166 |
|  | OR (95% CI) | OR (95% CI) | OR (95% CI) | OR (95% CI) [IOR-80] |
| ***Fixed effects at the individual level*** | | | | |
| *Intra-provincial migration (ref: never moved)* | | | | |
| Urban-Urban |  | 1.06 (0.70 - 1.60) | 2.03** (1.20 - 3.45) | 2.00** (1.20 - 3.35) |
| Rural-Rural |  | 1.35 (0.93 - 1.96) | 1.49* (1.01 - 2.19) | 1.49* (1.01 - 2.20) |
| Urban-Rural |  | 0.83 (0.59 - 1.19) | 1.21 (0.74 - 1.98) | 1.20 (0.73 - 1.96) |
| Rural-Urban |  | 1.29 (0.94 - 1.78) | 1.46 (0.98 - 2.17) | 1.45 (0.97 - 2.17) |
| *Inter-provincial migration (ref: never moved)* | | | | |
| Urban-Urban |  | 0.45** (0.25 - 0.81) | 0.87 (0.50 - 1.52) | 0.88 (0.50 - 1.53) |
| Rural-Rural |  | 1.37 (0.86 - 2.17) | 1.60* (1.09 - 2.34) | 1.63* (1.12 - 2.37) |
| Urban-Rural |  | 0.77 (0.51 - 1.17) | 1.32 (0.80 - 2.18) | 1.33 (0.82 - 2.17) |
| Rural-Urban |  | 0.89 (0.62 - 1.27) | 1.00 (0.68 - 1.49) | 1.01 (0.69 - 1.49) |
| Female-headed household *(ref: male-headed)* | |  | 1.16 (0.92 - 1.45) | 1.16 (0.92 - 1.45) |
| ***Fixed effects at the province level*** | | | | |
| Flooding (ref: no flooding) |  |  |  | 1.09 (0.77 - 1.54) [0.69 - 1.71] |
| Poorer province (ref: richer) |  |  |  | 1.30 (0.89 - 1.89) [0.83 – 2.04] |
| *Socio-geographic zones (ref: coastal)* | | | | |
| Plain |  |  |  | 1.44 (0.72 - 2.87) [0.92 – 2.26] |
| Plateau |  |  |  | 1.58 (0.74 - 3.39) [1.01 - 2.48] |
| Tonle Sap |  |  |  | 1.16 (0.57 - 2.37) [0.74 - 1.82] |
| SEZ |  |  |  | 0.98 (0.92 - 1.04) [0.63 - 1.54] |
| Constant | 0.29 (0.23 - 0.35) | 0.28 (0.22 - 0.35) | 0.06 (0.03 - 0.12) | 0.04 (0.01 - 0.11) |
| ***Random effects*** |  |  |  |  |
| ICC | 5.8% (3.1%-10%) | 6.0% (3.2%-10.9%) | 4.3% (1.8%-9.8%) | 1.8% (0.7%-5.0%) |
| MOR | 1.54 (1.32-1.75) | 1.55 (1.32-1.77) | 1.44 (1.21-1.68) | 1.27 (1.11-1.42) |

**** p<0.001, ** p<0.01, * p<0.05; OR, Odds Ratio; CI, Confidence Interval*; *IOR-80, Interval Odds Ratio at 80%; ICC, Interclass Correlation; MOR, median odds ratio. Model 0 was the null model with no explanatory variable. Subsequent models build upon it and each other by adding more factors. Model 1 included migration patterns. Model 2 added an interaction term between migration patterns and household-head gender and adjusted for confounders: education, individual wealth, urbanisation, household size, age, gender, marital status, and ethnicity. Model 3 added province-level factors such as flooding, provincial wealth, socio-geographic zones, and special economic zones (SEZ).*

**Table S7 Sensitivity analysis of associations between domestic migration patterns and household-head gender with food insecurity – using a multinomial model**

|  | Model 1 | Model 1 | Model 2 | Model 2 |
| --- | --- | --- | --- | --- |
| Chosen food insecurity level  *(ref: no food insecurity)* | Some food insecurity | Severe food insecurity | Some food insecurity | Severe food insecurity |
| Respondents | 5,166 | 5,166 | 5,166 | 5,166 |
|  | OR (95% CI) | OR (95% CI) | OR (95% CI) | OR (95% CI) |
| ***Fixed effects at the individual level*** | | | | |
| *Intra-provincial migration (ref: never moved)* | | | | |
| Urban-Urban | 0.77 (0.44 - 1.33) | 0.99 (0.58 - 1.68) | 1.15 (0.64 - 2.04) | 1.79* (1.01 - 3.19) |
| Rural-Rural | 1.45** (1.10 - 1.92) | 1.53** (1.16 - 2.02) | 1.50** (1.12 - 2.02) | 1.69*** (1.25 - 2.28) |
| Urban-Rural | 0.96 (0.55 - 1.66) | 0.83 (0.46 - 1.49) | 1.13 (0.64 - 2.00) | 1.12 (0.60 - 2.08) |
| Rural-Urban | 1.37 (0.94 - 1.99) | 1.43 (0.98 - 2.08) | 1.58* (1.06 - 2.36) | 1.74** (1.15 - 2.64) |
| *Inter-provincial migration (ref: never moved)* | | | | |
| Urban-Urban | 0.36*** (0.23 - 0.57) | 0.37*** (0.24 - 0.57) | 0.53* (0.33 - 0.86) | 0.73 (0.45 - 1.18) |
| Rural-Rural | 1.37* (1.07 - 1.76) | 1.52** (1.17 - 1.97) | 1.39* (1.07 - 1.81) | 1.66*** (1.25 - 2.21) |
| Urban-Rural | 1.00 (0.66 - 1.50) | 0.77 (0.49 - 1.22) | 1.18 (0.77 - 1.82) | 1.06 (0.65 - 1.74) |
| Rural-Urban | 0.81 (0.61 - 1.06) | 0.84 (0.63 - 1.11) | 1 (0.73 - 1.36) | 1.14 (0.82 - 1.58) |
| Female-headed household *(ref: male-headed)* | |  | 1.24* (1.02 - 1.50) | 1.19 (0.97 - 1.45) |
|  |  |  |  |  |
| Constant | 0.40*** (0.37 - 0.44) | 0.39*** (0.32 - 0.48) | 0.10*** (0.05 - 0.20) | 0.06*** (0.03 - 0.13) |

**** p<0.001, ** p<0.01, * p<0.05; OR, Odds Ratio; CI, Confidence Interval. Model 0 was the null model with no explanatory variable. Subsequent models build upon it and each other by adding more factors. Model 1 included migration patterns. Model 2 added an interaction term between migration patterns and household-head gender and adjusted for confounders: education, individual wealth, urbanisation, household size, age, gender, marital status, and ethnicity. Results from Model 3, which added province-level factors such as flooding, provincial wealth, socio-geographic zones, and special economic zones (SEZ), are not shown as convergence was not achieved. The distribution of each food insecurity category was: no food insecurity (n=2,832; 55%), some food insecurity (n=1,140; 22%), severe food insecurity (n=1,194; 23%).*

**Table S8 Effect size of the interaction term between migration pattern and the household-head gender from Model 3**

|  | | Male-headed households | | Female-headed households |
| --- | --- | --- | --- | --- |
| Migration patterns | | OR (95% CI) | | OR (95% CI) |
| Never moved | | 1 (reference) | | **1.23**** (1.08 - 1.40) |
| *Intra-provincially* | |  | |  |
| Urban-Urban | | 1.62 (0.91 - 2.88) | | 0.73 (0.18 - 2.96) |
| Rural-Rural | | **1.65**** (1.15 - 2.37) | | 1.74 (0.98 - 3.07) |
| Urban-Rural | | 1.19 (0.73 - 1.94) | | 1.07 (0.30 - 3.76) |
| Rural-Urban | | 1.42 (0.99 - 2.04) | | **3.69**** (1.68 - 8.10) |
| *Inter-provincially* | |  | |  |
| Urban-Urban | | 0.68 (0.41 - 1.13) | 0.68 (0.32 - 1.41) |  |
| Rural-Rural | | **1.59**** (1.15 - 2.19) | 1.75 (0.94 - 3.23) |  |
| Urban-Rural | | 1.23 (0.82 - 1.84) | 1.01 (0.45 - 2.23) |  |
| Rural-Urban | | 1.04 (0.79 - 1.36) | 1.59 (0.99 - 2.55) |  |

**** p<0.001, ** p<0.01, * p<0.05; OR, Odds Ratio; CI, Confidence Interval. Model 3 includes individual-level factors of migration patterns and their interaction with household-head gender, individual-level confounders: education, individual wealth, urbanisation, household size, age, gender, marital status, and ethnicity, and province-level factors: flooding, provincial wealth, socio-geographic zones, and special economic zones.*

**Table S9 Predicted probabilities of experiencing food insecurity by migration pattern and household-head gender from Model 3**

|  | Male-headed households | |  | Female-headed households | |
| --- | --- | --- | --- | --- | --- |
| Migration patterns | Predicted Probabilities | (95% CI) |  | Predicted Probabilities | (95% CI) |
| Never moved | 42.0% | (39.3 - 44.7) |  | 46.6% | (43.4 - 49.9) |
| *Intra-provincial migration* | |  |  |  |  |
| Urban-Urban | 52.8% | (39.9 - 65.7) |  | 35.3% | (6.6 - 64.0) |
| Rural-Rural | 53.2% | (46.3 - 60.1) |  | 54.3% | (42.8 - 65.9) |
| Urban-Rural | 45.9% | (34.9 - 56.9) |  | 43.5% | (15.1 - 71.8) |
| Rural-Urban | 49.9% | (41.2 - 58.5) |  | 70.0% | (55.2 - 84.8) |
| *Inter-provincial migration* | |  |  |  |  |
| Urban-Urban | 33.8% | (24.0 - 43.7) |  | 33.7% | (19.6 - 47.7) |
| Rural-Rural | 52.3% | (46.0 - 58.6) |  | 54.5% | (41.5 - 67.4) |
| Urban-Rural | 46.6% | (38.7 - 54.4) |  | 42.1% | (25.8 - 58.5) |
| Rural-Urban | 42.8% | (37.2 - 48.4) |  | 52.4% | (42.2 - 62.5) |

**** p<0.001, ** p<0.01, * p<0.05; OR, Odds Ratio; CI, Confidence Interval. Model 3 includes individual-level factors of migration patterns and their interaction with household-head gender, individual-level confounders: education, individual wealth, urbanisation, household size, age, gender, marital status, and ethnicity, and province-level factors: flooding, provincial wealth, socio-geographic zones, and special economic zones.*

**Table S10 Sensitivity analyses using all districts as the second level (A) and only districts with more than one outcome observation (B), instead of provinces.**

|  | Respondents | Districts | Observations per group; min (average) |  | Model 0 (95% CI) | Model 1 (95% CI) | Model 2 (95% CI) |
| --- | --- | --- | --- | --- | --- | --- | --- |
| A | 5,166 | 147 | 8 (35.1) | ICC | 14.4% (10.8%-19.0%) | 14.1% (10.4%-18.7%) | 12.0% (8.3%-17.1%) |
|  |  |  |  | MOR | 2.03 (1.80-2.27) | 2.01 (1.77-2.26) | 1.89 (1.64-2.15) |
|  |  |  |  |  |  |  |  |
| B | 5,041 | 139 | 12 (36.3) | ICC | 11.1% (8.6%-14.3%) | 10.8% (8.2%-14.1%) | 9.1% (6.4%-12.8%) |
|  |  |  |  | MOR | 1.84 (1.68-2.01) | 1.83 (1.66-1.99) | 1.73 (1.55-1.91) |

*ICC, Interclass Correlation; MOR, median odds ratio. Model 0 was the null model with no explanatory variable. Subsequent models build upon it and each other by adding more factors. Model 1 included migration patterns. Model 2 included an interaction term between the household-head gender and migration patterns and adjusted for confounders: education, individual wealth, urbanisation, household size, age, gender, marital status, and ethnicity.*
