# Supplementary material for: Food insecurity vulnerability among domestic migrants across Cambodian provinces: a multilevel analysis
Source: BMJ Public Health. 2026 Mar 4;4(1):e003281. doi: 10.1136/bmjph-2025-003281 (PMC12970070; doi:10.1136/bmjph-2025-003281)
Supplement: online supplemental file 2 [file bmjph-4-1-s002.pdf]

Supplementary Data D1. Contextual factors (province level data)

	**Variables**			
	**flooding**	**socio_geographic_zones**	**provincial_wealth** (population proportion within the national lowest wealth quintile)	**sez_num**
**Data sources for the variables**	Humanitarian Response Forum: Severe flooding in Sept - Oct 2022	Cambodia Agricultural Survey 2020	Cambodia Demographic and Health Survey 2021–22	Open Development Cambodia
**Links**	https://reliefweb.int/report/cambodia/floods-cambodia-2022-situation-report-sitrep-no-2-12-october-2022-humanitarian-response-forum-hrf	nis.gov.kh/nis/Agriculture/CAS 2020/Statistical Release CAS 2020_EN.pdf	https://dhsprogram.com/pubs/pdf/FR377/FR377.pdf	https://data.opendevelopmentcambodia.net/dataset/specialeconomiczoneincambodia20062014

**province_name**	**flooding**	**socio_geographic_zones**	**provincial_wealth** (population proportion within the national lowest wealth quintile)	**sez_num**
Phnom Penh	0	Plain	0	2
Kandal	0	Plain	3	4
Kep	0	Coastal	6.7	0
Preah Sihanouk	0	Coastal	7.5	12
Takeo	0	Plain	9	1
Prey Veng	0	Plain	11.8	0
Svay Rieng	0	Plain	13.4	11
Kampot	0	Coastal	15.4	2
Banteay Meanchey	1	Tonle Sap	17.7	3
Kampong Speu	1	Pleateau	17.9	2
Kampong Cham	1	Plain	19.9	1
Pailin	0	Tonle Sap	23.6	0
Koh Kong	0	Coastal	24.5	8
Tboung Khmum	1	Plain	25	0
Battambang	1	Tonle Sap	25.2	0
Siem Reap	1	Tonle Sap	28.1	0
Kampong Chhnang	1	Tonle Sap	34.1	1
Kampong Thom	1	Tonle Sap	34.3	0
Pursat	1	Tonle Sap	37.2	1
Otdar Meanchey	1	Tonle Sap	45.4	0
Kracheh	1	Pleateau	48.7	1
Mondul Kiri	1	Pleateau	51.4	0
Preah Vihear	1	Pleateau	60.9	0
Stung Treng	1	Pleateau	68.2	0
Ratanak Kiri	0	Pleateau	75.4	1
